# Prediction of the *n*-Octanol/Water Partition Coefficients of Basic Compounds Using Multi-Parameter QSRR Models Based on IS-RPLC Retention Behavior in a Wide pH Range

**DOI:** 10.3390/molecules28052270

**Published:** 2023-02-28

**Authors:** Jun-Qin Qiao, Xiao-Lan Liu, Chao Liang, Ju Wang, Hong-Zhen Lian, Li Mao

**Affiliations:** 1 State Key Laboratory of Analytical Chemistry for Life Science, School of Chemistry & Chemical Engineering and Center of Materials Analysis, Nanjing University, Nanjing 210023, China; 2 Taizhou Medical High-Tech Industrial Zone Public Platform Service Center, Taizhou 225300, China; 3 Nanjing Zhulu Pharmaceutical Technology Co., Ltd., Nanjing 211500, China; 4 Ministry of Education (MOE) Key Laboratory of Modern Toxicology, School of Public Health, Nanjing Medical University, Nanjing 211166, China

**Keywords:** ion-suppression reversed-phase liquid chromatography (IS-RPLC), basic compounds, apparent *n*-octanol/water partition coefficient (log*D*), quantitative structure–retention relationship (QSRR), multi-parameter models

## Abstract

The *n*-octanol–water partition coefficient (log*P*) is an important physicochemical parameter which describes the behavior of organic compounds. In this work, the apparent *n*-octanol/water partition coefficients (log*D*) of basic compounds were determined using ion-suppression reversed-phase liquid chromatography (IS-RPLC) on a silica-based C18 column. The quantitative structure–retention relationship (QSRR) models between log*D* and log*k*_w_ (logarithm of retention factor corresponding to 100% aqueous fraction of mobile phase) were established at pH 7.0–10.0. It was found that log*D* had a poor linear correlation with log*k*_w_ at pH 7.0 and pH 8.0 when strongly ionized compounds were included in the model compounds. However, the linearity of the QSRR model was significantly improved, especially at pH 7.0, when molecular structure parameters such as electrostatic charge *n*_e_ and hydrogen bonding parameters A and B were introduced. External validation experiments further confirmed that the multi-parameter models could accurately predict the log*D* value of basic compounds not only under strong alkaline conditions, but also under weak alkaline and even neutral conditions. The log*D* values of basic sample compounds were predicted based on the multi-parameter QSRR models. Compared with previous work, the findings of this study extended the pH range for the determination of the log*D* values of basic compounds, providing an optional mild pH for IS-RPLC experiments.

## 1. Introduction

Anilines, pyridines, imidazoles, triazines, and other basic compounds are essential chemical raw materials for national economies [1,2,3]. They are important organic intermediates in the production of vitamins, enzymes, and sulfonamides in pharmaceutical research. However, their production and use, as well as accidents arising from their storage and transportation, often cause these compounds to enter the environment, resulting in the pollution of air, soil/sediment, and water systems. These environmental risks also negatively affect wildlife [4]. At present, the environmental behavior of basic compounds is attracting considerable attention. The physicochemical properties of organic compounds determine the distribution and the fate of these pollutants in environmental media. Therefore, the determination of the physicochemical properties of basic compounds will help in assessing their potential environmental and health risks.

The *n*-octanol/water partition coefficient *P* (generally expressed as the logarithm form of *P*, log*P*), denotes the distribution of a chemical’s concentration in octanol and water when the octanol–water system is at equilibrium [5]. log*P* is a widely used and crucial parameter in investigating the fate of organic pollutants in the environment [6], and it remains a fundamental parameter for estimating bioaccumulation. The classical methods for log*P* determination are the shake-flask method (SFM) and the slow-stirring method (SSM) [7,8,9]. However, due to the great difficulty, time cosuming, and high cost inherent in SFM and SSM, the log*P* values of basic compounds obtained by these two methods are lacking. As a result, researchers have been committed to the development and improvement of calculation methods for determining log*P*, based on fragmental constants or an atomic contribution approach. In recent years, many calculation models have been developed and reported in the literature, such as the extreme learning machine model [5], the density functional theory (DFT) method [10], the norm index-based model [11], etc. These calculation methods are efficient for neutral compounds, but there are problems and limitations when applied to ionizable molecules, for which complex considerations are necessary [12,13,14]. Large deviations have been found between the calculated values and experimental values of ionizable compounds, especially those with complex structures [15]. Therefore, a technique for determining the log*P* values of basic compounds based on experimental methods is necessary.

High performance liquid chromatography (HPLC) is one method recommended by the Organisation for Economic Co-operation and Development (OECD) for log*P* determination [16]. Compared with SFM and SSM, HPLC is fast, consumes a low number of samples, and has a high level of automation. A significant advantage of HPLC is that the samples need not be of high purity [17]. Because of these distinct advantages, HPLC is widely used for the determination of the log*P* values or apparent *n*-octanol/water partition coefficients (log*D*) of organic compounds [17,18,19,20]. Among ionized compounds, the log*P* or log*D* values of acid compounds have been extensively studied, but those of basic compounds have been relatively ignored. In our previous work, Qi et al. [21] investigated the retention behavior of weakly ionized basic compounds on silica-based C18 columns using ion-suppression reversed-phase liquid chromatography (IS-RPLC) and established a linear relationship between log*D* and log*k*_w_ (logarithm of the retention factor at 100% aqueous phase as mobile phase). Notably, in Qi’s work [21], a relatively high pH of 9.0–11.5 was used, at which the dissociation of basic compounds was almost inhibited. Although it is beneficial for the determination of the log*D* values of basic compounds, the silica gel of the stationary phase can easily be destroyed under strong alkaline conditions, and this is detrimental to the continuity of experiments and leads to an increase in experimental cost and time. Is the relatively mild pH which helps to protect the silica-based column also suitable for the determination of the log*D* values of basic compounds in a dissociated state? The answer is not clear. Therefore, the feasibility of predicting the log*D* values of basic compounds in a dissociated state is worth studying.

In this work, we systematically investigated the IS-RPLC retention behavior of 42 basic compounds, including anilines, pyridines, imidazoles, and triazines, on a silica-based C18 column from pH values of 7.0 to 10.0. Methanol was used as the organic modifier and phosphate buffer was selected as the ion-suppressor. The univariate linear log*D*–log*k*_w_ models and multi-parameter QSRR models were established based on multiple linear regression (MLR). The applicability of these two kinds of models under each pH value was evaluated based on the linear regression correlation coefficient (*R*^2^). The results showed that the multi-parameter QSRR model exhibited advantages for determining the log*D* values of ionized basic compounds in a wide pH range. Based on the multi-parameter models, the log*D* values of 15 alkaline compounds were predicted at virous pH values.

## 2. Results and Discussion

### 2.1. Establishment of logD–logk_w_ Models and Comparison with Previous Work

The retention behavior of the 42 basic compounds (Table 1) were investigated at different ratios of methanol (*φ* = 0.7–0.1, the interval was 0.1 or 0.05 based on the retention of the investigated solutes). The log*k* values were plotted versus *φ* for each solute at pH 7.0, pH 8.0, pH 9.0, and pH 10.0. A log*k*–*φ* relationship diagram of some model compounds, verification compounds, and sample compounds is shown in Figure S1. The results showed that the log*k* values of the investigated solutes all had a good relationship against *φ* at each pH value, with a linear correlation coefficient *R*^2^ greater than 0.99 in each case. This phenomenon confirmed that the retention behavior of ionizable compounds still satisfies the linear solvent strength (LSS) model (Equation (S1)) in IS-RPLC. The log*k*_w_ value of each compound was then obtained using Equation (S1), and the values are summarized in Table 1. The log*D* values of the model compounds and verification compounds calculated using Equation (S3) based on the log*P* and pH values are also listed in Table 1.

**Figure 1:**
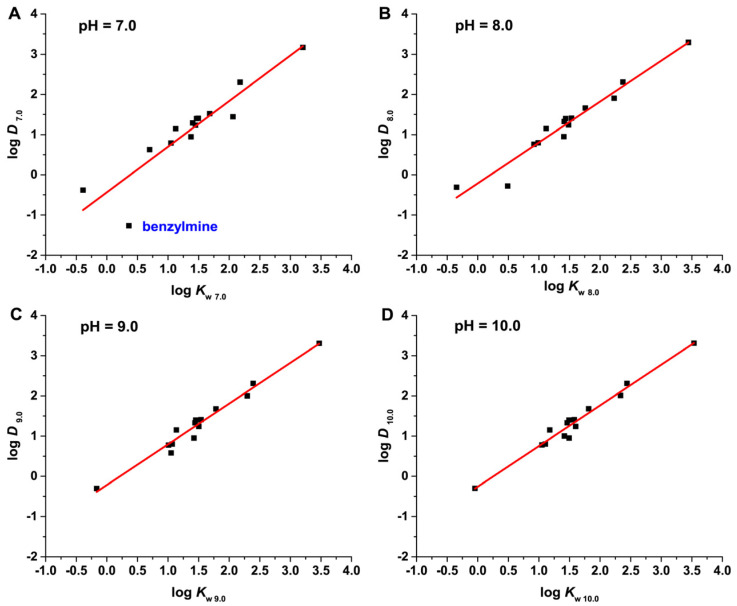
Linear plots of log*D* versus log*k*_w_ for model compounds **1**–**14** at different mobile phase pH values. (**A**) pH = 7.0; (**B**) pH = 8.0; (**C**) pH = 9.0; (**D**) pH = 10.0.

Notably, the values of the model compounds **1**–**14** were consistent with those reported in Qi’s work [21]. For these 14 compounds, the log*D* was plotted against the log*k*_w_ and linearly fitted at a pH of 7.0–10.0 (Figure 1). As Figure 1 shows, the dots were relatively dispersed at pH 7.0. As a result, the linearity of the log*D*–log*k*_w_ models at this pH was poor, with *R*^2^ values of only 0.825, as is shown in Table 2. When the pH was raised to 8.0–10.0, the dots became more and more concentrated, with a significantly improved linearity of the models (*R*^2^ = 0.943–0.969). Additionally, the *R*^2^ value at pH 9.0 and pH 10.0 were almost the same, suggesting that the linearity of the models did not change much after pH 9.0. This may be attributed to the fact that the dissociation of most of the model compounds was well inhibited at pH 9.0 and above, leading to a similarity in the retention behavior of the solutes.

By comparing Figure 1A–D, it was found that the poor linearity at pH 7.0 was mainly caused by the deviation of benzylamine from other points. In structure, benzylamine is different from aniline and pyridine. The lone pair of N atoms in benzylamine is not coplanar, but at an angle of about 50° with the $\pi_{6}^{6}$ of the benzene ring. The saturated methylene prevents the lone pair from entering the benzene ring for conjugation, resulting in the strong dissociation of the amino group in benzylamine. It is known that the dissociable basic compounds form cations of the corresponding acid at pH < p*K*_a_, and that the dissociation can be completely inhibited only when the mobile phase pH ≥ p*K*_a_ + 2. As Table 1 shows, the p*K*_a_ (9.35) of benzylamine was significantly higher than those of the other 13 model compounds. Therefore, the benzylamine was almost completely dissociated at pH 7.0, leading to a relatively different retention behavior compared with the other 13 compounds. With the increased pH of the mobile phase, the dissociation of the benzylamine was partially suppressed, and the difference in retention behavior compared with the other compounds gradually decreased. If benzylamine was removed from the model compounds, the linear correlation of the log*D*–log*k*_w_ models at all pH values would improve, especially at pH 7.0 (Table S1).

A comparation of the log*D*–log*k*_w_ models in this work with those in Qi’s work [21] was conducted. It was found that the linear models obtained in this work were better than those obtained in Qi’s work at pH 9.0 and pH 10.0. There are three possible reasons for this: First, the different ion-suppressors in these two works. In Qi’s work, ammonia and triethylamine (TEA) were used as ion-suppressors, but in this work, ammonium phosphate buffer was used as the ion-suppressor. Second, the improvement in column performance. The current chromatographic columns are always superior to those used in the past, both in terms of packing technology and packing composition, which is beneficial for obtaining a good peak and the accurate retention of the solutes. Third, the introduction of the dual-point retention time correction (DP-RTC) method [22] in this work. Research has shown that the retention time correction method can make the acquirement of retention time more accurate [22]. To better understand which was the main reason for the different linearities of the models in these two works, model compounds 1–14 were further investigated on the Welch Xtimate C18 column using ammonia and triethylamine (TEA) as ion-suppressors at pH 9.0 (the same ion-suppressors used in Qi’s work). The resulting log*D*–log*k*_w_ models and linear regression coefficients are shown in Table S2. Obviously, under the same mobile phase, the linearities of the log*D*–log*k*_w_ models on the Welch Xtimate C18 column were superior to those on the Phenomenex Gemini C18 column, especially with TEA as an ion-suppressor. Therefore, the better performance of the current column and the introduction of the DP-RTC method both contributed to the improved linearity of the log*D*–log*k*_w_ models in this work.

It was further discovered that, on the Welch Xtimate C18 column at pH 9.0 with TEA as an ion-suppressor, the log*D*–log*k*_w_ linear correlation (*R*^2^ = 0.961, Table S2) was almost equal to that obtained with phosphate buffer as an ion-suppressor (*R*^2^ = 0.968, Table 2). However, the log*D*–log*k*_w_ linear correlation was relatively low when using ammonia as an ion-suppressor (*R*^2^ = 0.931), though it was still higher than that obtained in Qi’s work (*R*^2^ = 0.925). It maybe that the ionic strength of the ammonia solution was lower than that of the triethylamine solution and the phosphate buffer, and that affected the retention behavior of the solutes. Since phosphate buffer can not only inhibit the dissociation of solutes, but can also maintain the ion strength of the mobile phase, it was adopted in the following experiments in this work.

### 2.2. Establishment of Multi-Parameter QSRR Models

As mentioned above, when compounds with high p*K*_a_ values were used as model compounds, the log*D*–log*k*_w_ linearity was not good at low pH, and this was unfavorable for accurately predicting the log*D* values of strong ionized alkaline compounds. How can this problem be solved? As we know, the more model compounds, the better the linearity of the models will be, resulting in more accurate log*D* predictions. Therefore, in addition to the above 14 compounds, a further 9 compounds (No. 15–23, Table 1) with experimental log*P* and p*K*_a_ values were introduced as model compounds. Altogether, these are the compounds 1–23 listed in Table 1. Similarly, log*D* values were plotted versus log*k*_w_ values, and the corresponding log*D*–log*k*_w_ models are listed in Table S3.

Contrary to our expectations, the linear correlation of the log*D*–log*k*_w_ models was not obviously improved compared with that listed in Table 2. In the study of acidic ionized compounds, we proposed that the involvement of molecular structure parameters such as electrostatic charge *n*_e_, hydrogen bonding parameter A, and hydrogen bonding parameter B could effectively improve the correlation of the log*D*–log*k*_w_ models [15,19,23]. Inspired by this, we speculated that this rule may also be applicable to ionized basic compounds. Thus, the parameters *n*_e_, A, and B were introduced to the models, and the values of the three parameters for all the investigated compounds are listed in Table S4.

Multi-parameter QSRR models including log*D*, log*k*_w_, *n*_e_, A, and B were established using the multiple linear regression (MLR) method. In the process of linear fitting, *n*_e_, A, B, and their different combinations were introduced to optimize the models. It was found that, at pH 7.0 and pH 8.0, the models which included *n*_e_ exhibited the best linearity, suggesting that electrostatic interaction as well as hydrophobic interaction played an important role in solutes’ retention under neutral and weak alkaline conditions. Because at pH 7.0 and pH 8.0 (though especially at pH 7.0) some model compounds were in a state of completed dissociation and some in a state of partial dissociation, there was strong electrostatic interaction between the ionized solutes and the stationary phase and mobile phase. In contrast, at pH 9.0 and pH 10.0, the linearity was the best when *n*_e_, A, and B were all introduced at the same time. This suggests that both electrostatic interaction and hydrogen bond interaction were the main secondary actions affecting the retention of the basic compounds under relatively strong alkaline conditions when the dissociation was weak. The best multi-parameter QSRR models at each investigated pH are listed in Table 3. From Table 3, we can see that the linearity of the models improved significantly compared with the models that contained no molecular structure parameters, with an *R*^2^ value of 0.946 achieved at pH 7.0. It was thus proved that the multi-parameter QSRR models for the determination the log*D* values of basic compounds could be properly established under strong alkaline, weak alkaline, and even at neutral conditions.

### 2.3. External Validation of Multi-Parameter Models and Sample logD Determination

Furthermore, to evaluate the reliability and accuracy of the multi-parameter QSRR models, four compounds whose log*P* values have been reliably reported in the literature—4-bromoaniline, 2-ethylpyridine, 2-ethylaniline, and dibenzylamine—were chosen as the validation compounds to perform the external verification experiment. The log*D* values reported in the literature for these four compounds were calculated using Equation (S3) with corresponding p*K*_a_, log*P*, and pH values. The verification results are listed in Table 4. It was observed that the relative errors between the determined log*D* values (the values determined by the models in Table 3) and the literature log*D* values were all within the acceptable range of 20%, not only at high pH values of 9.0–10.0, but also at low pH values of 7.0–8.0. It was also found that the models were able to accurately predict the log*D* values of both weak basic compounds and strong ionized basic compounds. The validation results showed that the multi-parameter QSRR models established in this work have strong robustness, good predictability, and wide pH applicability.

Based on the multi-parameter models, the log*D* values of the 15 basic sample compounds, including aniline, imidazole, and triazine (No. 28–42 in Table 1), were predicted at pH 7.0, pH 8.0, pH 9.0, and pH 10.0. The determined log*D* results are given in Table 5. Notably, this is the first time that experimental log*D* values have been reported for triazines, as far as we know.

## 3. Materials and Methods

### 3.1. Materials

Methanol (HPLC grade) was purchased from Honeywell Burdick & Jackson (Muskegon, MI, USA). Ammonium phosphate (analytical-reagent grade), phosphoric acid (85%, analytical-reagent grade), and ammonia (25–28%, analytical-reagent grade) were all purchased from Nanjing Chemical Reagent Co., Ltd. (Nanjing, China). Triethylamine (HPLC grade) was purchased from TEDIA Co., Ltd. (Fairfield, OH, USA). The water used throughout was purified water (Wahaha Group, Hangzhou, China).

The investigated compounds, as well as their log*P* and p*K*_a_ values as reported in the literature, are all listed in Table 1. All the compounds were obtained from commercial sources (AccuStandard, New Haven, CT, USA; TCI, Tokyo, Japan; Sinopharm Chemical Reagent Co., Ltd., Shanghai, China; J&K Scientific Co., Ltd., Beijing, China; Acros Organics, NJ, USA; Matrix Scientific, Columbia, SC, USA; Sigma-Aldrich, St. Louis, MO, USA), and all of them had a purity of 98% or higher. In the experiments, these compounds were divided into the following three groups: model compounds, verification compounds, and sample compounds. Stock solutions of each solute at concentrations of about 1.0 mg/mL were prepared in methanol and stored in a refrigerator at 4 °C before use.

### 3.2. Instruments and Equipment

A Waters 2695 Alliance separation module (Milford, MA, USA) consisting of a vacuum degasser, a quaternary pump, an auto-sampler, and a Waters 996 photodiode-array detector was employed to perform the HPLC experiments.

The pH was measured with a SevenMulti electrochemical analytical meter (Metter-Toledo, Schwerzenbach, Switzerland). The electrode system was standardized with ordinary aqueous buffers of pH 4.01, pH 7.02, pH 9.26 and pH 11.00 at 25 °C. All pH readings were carried out in buffer solution.

### 3.3. Chromatographic Condition

The chromatographic column used in the experiment was a Welch Xtimate^®^ C18 (150 mm × 4.6 mm i.d., 5 µm, Welch Technology Co., Ltd., Shanghai, China) with an alkaline-resistant stationary phase, a pH range of 1.0–12.5, and a flow rate of 1.0 mL/min. The column temperature was set at 30 °C. Methanol and 20 mmol/L ammonium phosphate buffer (pH = 7.0, 8.0, 9.0, 10.0) was used as the mobile phase to perform isocratic elution. The injection volume was 10 µL and the detection wavelength for each eluted compound was set at its optimum absorption wavelength.

### 3.4. Experimental Methods

At each pH, the retention time (*t*_R_) of every analyte was determined for at least four different volume fractions (*φ*) of methanol. All the *t*_R_ values of the solutes were obtained by averaging the results of at least three independent injections. The *t*_R_ value was corrected using dual-point retention time correction (DP-RTC). For a detailed description of the process, refer to our previous work [23]. For the different hydrophobicities of the studied compounds, benzene, toluene, and benzyl alcohol were used as anchor compounds in the DP-RTC method. The dead time *t*_0_ was determined using uracil. For each solute, the logarithm of *k* was plotted against *φ*, and log*k*_w_ was obtained using the Equation (S1). The log*D* values of the model compounds were calculated using Equation (S3), and the log*D*–log*k*_w_ models were established with Equation (S4). The statistical analysis for the regression model was accomplished using Origin 9.4.

## 4. Conclusions

The retention behaviors of aniline, pyridine, imidazole, and triazine compounds were studied using IS-RPLC on a silica-based C18 column at pH 7.0–10.0. Multi-parameter QSRR models were established by introducing molecular structure parameters such as *n*_e_, A, and B for determining the log*D* values of basic compounds. It was proposed that the QSRR models had strong robustness, good predictability, and wide pH applicability because the log*D* values of ionized basic compounds could not only be accurately determined under strong alkaline conditions with the dissociation mostly inhibited, but they could also be determined under weak alkaline or even neutral conditions with solutes in an ionized state using the established models. Moreover, we successfully predicted the log*D* values of 15 basic sample compounds using the developed multi-parameter QSRR models at pH 7.0, pH 8.0, pH 9.0 and pH 10.0. This work made up for the deficiency of our previous work on the prediction of the log*D* values of basic compounds, providing an optional mild pH for experimental log*D* determination. Under relatively weak alkaline conditions or at neutral pH, it is not only convenient to adjust the pH of the mobile phase, but it is also important to protect the chromatographic column of the silica gel matrix, which helps to enhance the life of the silica-based columns and save money and time.

## Figures and Tables

**Table 1:** The log*P* and p*K*_a_ values of each of the investigated compounds, as reported in the literature, as well as the corresponding log*D* values and the determined log*k*_w_ values.

No.	Model Compound	log*P*	p*K*_a1_	p*K*_a2_	log*D*_pH_				log*k*_w-pH_			
					7.0	8.0	9.0	10.0	7.0	8.0	9.0	10.0
1	2-Methylaniline	1.40	4.45		1.40	1.40	1.40	1.40	1.47	1.44	1.45	1.49
2	4-Methylaniline	1.41	5.08		1.40	1.41	1.41	1.41	1.50	1.53	1.54	1.58
3	N, N-Diethylaniline	3.31	6.57		3.17	3.29	3.31	3.31	3.20	3.44	3.47	3.54
4	4-Methylpyridine	1.33	5.99		1.29	1.33	1.33	1.33	1.40	1.41	1.44	1.46
5	4-Fluoroaniline	1.15	4.65		1.15	1.15	1.15	1.15	1.13	1.12	1.14	1.18
6	2,6-Dimethylpyridine	1.68	6.65		1.52	1.66	1.68	1.68	1.68	1.76	1.78	1.81
7	2,4,6-Trimethylpyridine	2.01	7.43		1.44	1.91	2.00	2.01	2.06	2.23	2.30	2.34
8	N, N-Dimethylaniline	2.31	5.07		2.30	2.31	2.31	2.31	2.18	2.37	2.39	2.44
9	Benzylamine	1.09	9.35		−1.26	−0.28	0.58	1.00	0.36	0.49	1.05	1.42
10	4-Ethoxyaniline	1.24	5.25		1.23	1.24	1.24	1.24	1.45	1.49	1.50	1.60
11	2-Methoxyaniline	0.95	4.53		0.95	0.95	0.95	0.95	1.38	1.41	1.42	1.49
12	4-Methoxyaniline	0.80	5.36		0.79	0.80	0.80	0.80	1.05	0.99	1.07	1.11
13	1,4-Benzenediamine	−0.30	6.31	2.97	−0.38	−0.31	−0.30	−0.30	−0.39	−0.35	−0.17	−0.04
14	Pyridine	0.78	6.62		0.63	0.76	0.78	0.78	0.70	0.92	1.01	1.05
15	N, N-Dimethylbenzylamine	1.98	8.80		0.17	1.12	1.77	1.95	0.97	1.61	2.13	2.44
16	2-Amino-4-methylpyridine	0.89	7.38		0.36	0.80	0.88	0.89	1.00	1.16	1.28	1.32
17	4-Isopropylaniline	2.23	4.85		2.23	2.23	2.23	2.23	2.42	2.57	2.59	2.65
18	2,4-Dimethylpyridine	1.65	6.58		1.51	1.63	1.65	1.65	1.76	1.85	1.88	1.96
19	2,4-Dimethylaniline	1.68	4.70		1.68	1.68	1.68	1.68	1.92	1.98	2.00	2.04
20	2-Amino-6-methylpyridine	1.08	6.95		0.53	0.98	1.07	1.08	0.99	1.13	1.19	1.27
21	Aniline	0.90	4.60		0.90	0.90	0.90	0.90	0.96	0.95	0.97	1.02
22	4-Phenylpyridine	2.59	5.45		2.58	2.59	2.59	2.59	2.66	2.81	2.84	2.80
23	2-Picoline	1.09	5.94		1.05	1.09	1.09	1.09	1.31	1.32	1.34	1.36
**No.**	**Verification Compounds**											
24	4-Bromoaniline	2.05	3.89		1.74	1.74	1.74	1.74	1.91	1.98	1.99	1.99
25	2-Ethylaniline	1.74	4.37		1.63	1.67	1.67	1.67	1.87	1.94	1.95	1.95
26	2-Ethylpyridine	1.67	5.97		2.05	2.05	2.05	2.05	1.74	1.79	1.80	1.90
27	Dibenzylamine	3.03	8.76		1.26	2.20	2.83	3.01	2.42	2.99	3.28	4.54
**No.**	**Sample Compounds**											
28	1,2-diaminobenzene	NA							0.50	0.49	0.29	0.60
29	1,3-diaminobenzene	NA							0.17	0.07	−0.16	0.21
30	2-Methyl-4-nitroaniline	NA							1.76	1.79	1.46	1.84
31	2,4-Dinitroaniline	NA							1.80	1.86	1.78	1.92
32	2-Chloro-4-nitroaniline	NA							2.07	2.13	2.14	2.18
33	2-Chloro-4,6-dinitroaniline	NA							2.19	2.25	2.26	2.30
34	1,1’-Carbonyldiimidazole	NA							−0.39	−0.38	−0.27	−0.15
35	Etiracetam	NA							0.80	0.66	0.82	0.88
36	2-Amino-4-methyl-6-methoxy-s-triazine	NA							1.36	1.36	1.38	1.43
37	Citrazinic acid	NA							1.18	1.18	1.20	1.30
38	2-Amino-1,3,5-triazine	NA							1.36	1.36	1.38	1.43
39	4-Iodoaniline	NA							2.22	2.28	2.29	2.28
40	Imidazole	NA							−0.53	−0.39	−0.34	−0.16
41	4-Methylimidazole	NA							−0.08	0.19	0.32	0.39
42	3,3’-Sulfonyldianiline	NA							1.78	1.83	1.83	1.47

The literature log*P* and p*K*_a_ values were obtained from the database module of ACD/Labs software; The log*D* values were calculated with log*P*, p*K*_a_, and pH using Equation (S3). NA: no log*P* value obtained from the literature.

**Table 2:** log*D*–log*k*_w_ relationships derived from model compounds **1**–**14** at different mobile phases.

	Buffer	pH	log*D*–log*k*_w_	*R^2^*
This work	Phosphate buffer	7.0	log*D* = (1.13 ± 0.14) log*k*_w_ − (0.44 ± 0.23)	0.825
		8.0	log*D* = (1.02 ± 0.07) log*k*_w_ − (0.21 ± 0.12)	0.943
		9.0	log*D* = (1.01 ± 0.05) log*k*_w_ − (0.22 ± 0.09)	0.968
		10.0	log*D* = (1.01 ± 0.05) log*k*_w_ − (0.26 ± 0.09)	0.969
Qi et al. [21]	Ammonia solution	9.0	log*D* = (1.07 ± 0.07) log*k*_w_ − (0.28 ± 0.12)	0.944
		10.0	log*D* = (1.01 ± 0.08) log*k*_w_ − (0.19 ± 0.13)	0.928
	TEA solution	9.0	log*D* = (1.00 ± 0.07) log*k*_w_ − (0.14 ± 0.12)	0.935
		10.0	log*D* = (1.05 ± 0.07) log*k*_w_ − (0.15 ± 0.12)	0.941

**Table 3:** Multi-parameter QSRR models derived from the 23 model compounds.

pH	log*D*–log*k*_w_	*N*	*R* ^2^
7.0	log*D* = (1.02 ± 0.06) log*k*_w_ − (0.85 ± 0.14) *n*_e_ − (0.12 ± 0.12)	23	0.946
8.0	log*D* = (0.96 ± 0.04) log*k*_w_ − (0.54 ± 0.10) *n*_e_ − (0.09 ± 0.06)	23	0.976
9.0	log*D* = (0.93 ± 0.04) log*k*_w_ − (0.29 ± 0.17) *n*_e_ + (0.21 ± 0.24) A − (0.49 ± 0.26) B + (0.21 ± 0.20)	23	0.976
10.0	log*D* = (0.92 ± 0.04) log*k*_w_ − (0.67 ± 0.78) *n*_e_ − (0.30 ± 0.24) A − (0.62 ± 0.26) B + (0.26 ± 0.20)	23	0.978

**Table 4:** External verification of multi-parameter QSRR models at different pH values.

Compound	p*K*_a_	log*P*	pH	Literature log*D*	Determined log*D*	Error (%)
4-Bromoaniline	3.89	2.05	7.0	2.05	1.83	−10.73
			8.0	2.05	1.81	−11.71
			9.0	2.05	1.85	−9.76
			10.0	2.05	1.81	−11.71
2-Ethylaniline	4.37	1.74	7.0	1.74	1.79	2.87
			8.0	1.74	1.77	1.72
			9.0	1.74	1.75	0.57
			10.0	1.74	1.71	−1.72
2-Ethylpyridine	5.97	1.67	7.0	1.63	1.62	−0.61
			8.0	1.67	1.62	−2.99
			9.0	1.67	1.60	−4.19
			10.0	1.67	1.65	−1.20
Dibenzylamine	8.76	3.03	7.0	1.26	1.52	20.63
			8.0	2.20	2.36	7.27
			9.0	2.83	2.81	−0.71
			10.0	3.00	3.00	0

**Table 5:** log*D* values of the sample compounds at pH 7.0, pH 8.0, pH 9.0, and pH 10.0 determined using IS-RPLC.

Sample Compound	log*D*_7.0_	log*D*_8.0_	log*D*_9.0_	log*D*_10.0_
o-Phenylenediamine	0.38	0.38	0.07	0.28
m-Phenylenediamine	0.03	−0.02	−0.41	−0.15
2-Methyl-4-nitroaniline	1.67	1.63	1.31	1.60
2,4-Dinitroaniline	1.71	1.69	1.58	1.64
2-Chloro-4-nitroaniline	1.99	1.96	1.93	1.92
2-Chloro-4,6-dinitroaniline	2.11	2.07	1.99	1.97
1,1’-Carbonyldiimidazole	−0.52	−0.46	−0.84	−0.89
Etiracetam	0.70	0.54	0.17	0.04
2-Amino-4-methyl-6-methoxy-s-triazine	1.28	1.22	0.94	0.86
Citrazinic acid	2.29	2.05	1.2	1.87
2-Amino-1,3,5-triazine	1.27	1.22	0.95	0.88
4-Iodoaniline	2.14	2.10	2.13	2.08
Imidazole	−1.07	−0.5	−0.58	−0.50
4-Methylimidazole	−0.54	0.06	0.08	0.07
3,3’-Sulfonyldianiline	1.70	1.67	1.20	0.69

## Data Availability

Not applicable.

## Supplementary Materials

The following supporting information can be downloaded at: https://www.mdpi.com/article/10.3390/molecules28052270/s1, Figure S1: The linear plots of log*k* versus *φ* of some model compounds (**1**, **2**, **3**, **4**, **5**), verification compounds (**24**, **25**) and sample compounds (**28**, **29**, **30**) at different mobile phases, Table S1: log*D*–log*k*_w_ relationships derived from 13 model compounds at different mobile phase pH values, Table S2: log*D*–log*k*_w_ models derived from 14 model compounds with different ion-suppressors and columns, Table S3: log*D*–log*k*_w_ models derived from 23 model compounds at different mobile phase pH values, Table S4: Molecular structure parameter values of *n*_e_, A, and B for all the investigated compounds [24,25,26].

## Sample Availability

Samples of the compounds are not available from the authors.
