# Supplementary material for: Prediction of the n-Octanol/Water Partition Coefficients of Basic Compounds Using Multi-Parameter QSRR Models Based on IS-RPLC Retention Behavior in a Wide pH Range
Source: Molecules. 2023 Feb 28;28(5):2270. doi: 10.3390/molecules28052270 (PMC10005301; doi:10.3390/molecules28052270)
Supplement: Supplementary file 1 [file molecules-28-02270-s001.zip › molecules-2234605-supplementary.pdf]

Supporting Information

Prediction of *n*-Octanol/Water Partition Coefficients of Basic Compounds by Multi-parameter QSRR Models Based on IS-RPLC Retention Behaviour in Wide pH

Jun-Qin Qiao [1], Xiao-Lan Liu [1,2], Chao Liang [1,3], Ju Wang [1], Hong-Zhen Lian [1,*] and Li Mao [4,*]

[1] State Key Laboratory of Analytical Chemistry for Life Science, School of Chemistry & Chemical Engineering and Center of Materials Analysis, Nanjing University, Nanjing 210023, China;

[2] Taizhou Medical High-Tech Industrial Zone Public Platform Service Center, Taizhou 225300, China;

[3] Nanjing Zhulu Pharmaceutical Technology Co., Ltd., Nanjing 211500, China

[4] Ministry of Education (MOE) Key Laboratory of Modern Toxicology, School of Public Health, Nanjing Medical University, Nanjing 211166, China

* Correspondence: hzlian@nju.edu.cn (H.-Z.L.); maoli@njmu.edu.cn (L.M.)

Theoretical basis

Studies have shown that logk_w could well describe the lipophilic index of compounds. The logk_w is usually obtained by linear solvent strength (LSS) model [24].

$$\log k = \log k_w - S\varphi \tag{S1}$$

Where, k is the capacity factor, φ is the volume fraction of organic modifier in the mobile phase, and S is the constant obtained by linear regression in the equations.

Both theory and experiments prove that there is a good linear relationship between log$P$ and logk_w, which is usually called Collander equation [25].

$$\log P = m \log k_w + n \tag{S2}$$

Where, m and n are constants obtained by linear regression.

The Eqs. (S1) and (S2) are the basis for the determination of log$P$ by revered-phase liquid chromatography (RPLC) method. For dissociative solutes, it has been confirmed by many studies that the log$D$ calibrated from log$P$ has a better linear correlation with logk_w than log$P$. For basic compounds, the log$D$ value can be calibrated from Eq. (S3), and the Collander equation can be rewritten into Eq. (S4).

$$\log D = \log\left(\frac{P}{1+10^{(\mathrm{p}K_{\mathrm{a_n}}-\mathrm{pH})}+\ldots+10^{(\mathrm{p}K_{\mathrm{a_n}}-\mathrm{pH})+(\mathrm{p}K_{\mathrm{a_{n-1}}}-\mathrm{pH})+\ldots+(\mathrm{p}K_{\mathrm{a_1}}-\mathrm{pH})}}\right) \tag{S3}$$

$$\log D = m' \log k_w + n' \tag{S4}$$

Where, pK_{a1}, pK_{a2}, … pK_{an} are the dissociation constants of basic compounds of corresponding acids in the mobile phase, pH is the pH value of mobile phase, m′ and n′ are fitting parameters. Han et al. [26] pointed out that although mobile phase pH and pK_a of solutes will change along with the varied organic modifier fraction, the difference between a couple of relevant pH and pK_a is a constant. Therefore, the used pH and pK_a in this paper were both the values obtained at 100% aqueous phase.

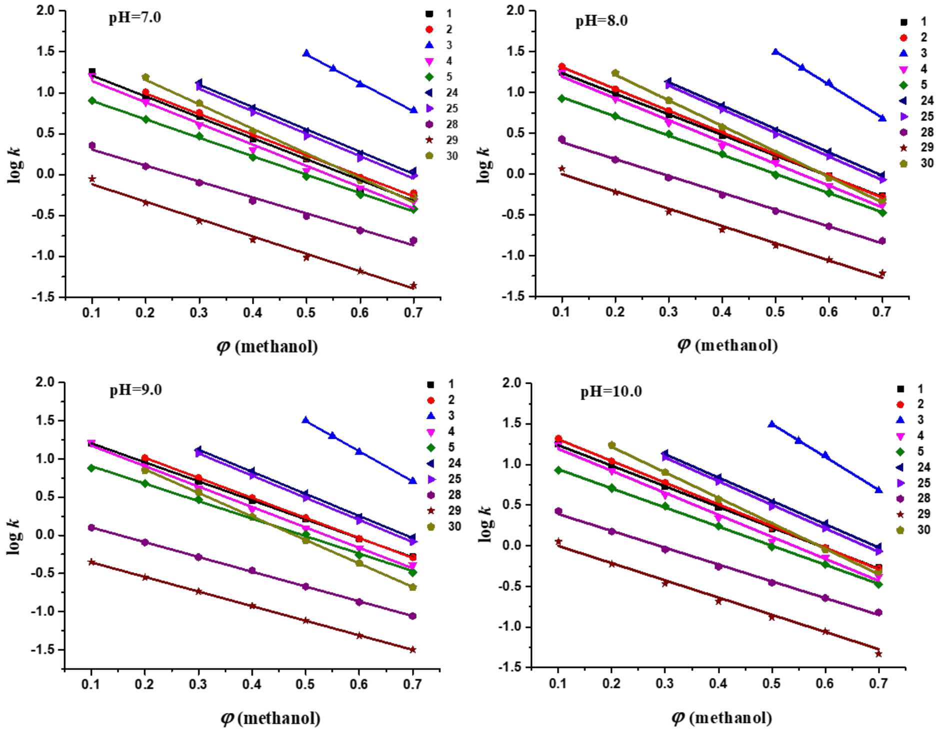

Figure S1. The linear plots of log k versus φ of some model compounds (1, 2, 3, 4, 5), verification compounds (24, 25) and sample compounds (28, 29, 30) at different mobile phases pH. φ was the ratio of methanol in mobile phase. 1, 2, 3, 4, 5, 24, 25, 28, 29, 30 corresponded to the compounds listed in Table 1.

Table S1. Relationships of log$D$-logk_w derived from 13 model compounds at different mobile phase pH values.

Buffer	pH	log$D$(y)-logk_w(x)	N	R^2
Phosphate buffer	7.0	y=0.973 x−0.107	13	0.9383
	8.0	y=0.956 x−0.077	13	0.9735
	9.0	y=0.997 x−0.172	13	0.9759
	10.0	y=1.007 x−0.240	13	0.9722

Table S2. log$D$-logk_w models derived from 14 model compounds with different ion-suppressors and columns.

	Ion-suppressor	Column	pH	log$D$(y)-logk_w(x)	N	R^2
This work	TEA solution	Welch Xtimate	9	y=0.892 x−0.042	14	0.961

	Ammonia solution	C18	9	y=1.016 x−0.309	14	0.931
Qi et al.	TEA solution	Phenomenex	9	y=1.003 x−0.143	14	0.936
work	Ammonia solution	Gemini C18	9	y=1.070 x−0.285	14	0.925

Table S3. log*D*-logk_w models derived from 23 model compounds at different mobile phase pH values.

Buffer	pH	log*D*-logk_w	*N*	R^2
Phosphate buffer	7.0	log*D*=(1.15±0.10) logk_w−(0.50±0.16)	23	0.857
	8.0	log*D*=(1.00±0.05) logk_w−(0.21±0.09)	23	0.944
	9.0	log*D*=(0.98±0.04) logk_w−(0.19±0.07)	23	0.967
	10.0	log*D*=(0.98±0.04) logk_w−(0.23±0.07)	23	0.966

Table S4. Values of molecular structure parameters of n_e, A and B for all the investigated compounds.

No.	Model compounds	n_e				A	B
		pH 7.0	pH 8.0	pH 9.0	pH 10.0		
1	2-Methylaniline	0.00	0.00	0.00	0.00	0.92	0.97
2	4-Methylaniline	0.01	0.00	0.00	0.00	0.91	0.94
3	N, N-Diethylaniline	0.07	0.01	0.00	0.00	0.80	0.95
4	4-Methylpyridine	0.07	0.01	0.00	0.00	0.82	0.63
5	4-Fluoroaniline	0.00	0.00	0.00	0.00	1.09	1.76
6	2,6-Dimethylpyridine	0.26	0.03	0.00	0.00	0.70	0.61
7	2,4,6-Trimethylpyridine	0.67	0.17	0.02	0.00	0.69	0.63
8	N, N-Dimethylaniline	0.01	0.00	0.00	0.00	0.81	0.96
9	Benzylamine	1.00	0.95	0.67	0.16	0.77	0.83
10	4-Ethoxyaniline	0.01	0.00	0.00	0.00	1.17	0.95
11	2-Methoxyaniline	0.00	0.00	0.00	0.00	1.00	0.99
12	4-Methoxyaniline	0.01	0.00	0.00	0.00	1.26	1.05
13	1,4-Benzenediamine	0.22	0.03	0.00	0.00	1.73	1.30
14	Pyridine	0.01	0.00	0.00	0.00	0.82	0.60
15	N, N-Dimethylbenzylamine	0.99	0.89	0.44	0.07	0.80	0.67
16	2-Amino-4-methylpyridine	0.81	0.29	0.04	0.00	1.09	0.95
17	4-Isopropylaniline	0.01	0.00	0.00	0.00	0.87	0.92
18	2,4-Dimethylpyridine	0.27	0.04	0.00	0.00	0.76	0.63
19	2,4-Dimethylaniline	0.01	0.00	0.00	0.00	0.95	0.95
20	2-Amino-6-methylpyridine	0.80	0.29	0.04	0.00	1.15	0.93
21	Aniline	0.00	0.00	0.00	0.00	1.08	1.86
22	4-Phenylpyridine	0.01	0.00	0.00	0.00	1.36	1.38
23	2-Picoline	0.06	0.01	0.00	0.00	0.75	0.60
**No.**	**Verification compounds**						
24	Dibenzylamine	0.97	0.78	0.26	0.03	1.27	1.30
25	2-Ethylaniline	26.00	0.23	0.45	0.00	0.85	0.96
26	2-Ethylpyridine	12.90	0.00	0.59	0.00	0.71	0.61
27	4-Bromoaniline	26.00	0.31	0.30	0.00	1.19	1.19

No.	Sample compounds						
28	1,2-diaminobenzene	52.00	0.24	0.73	1.00	1.40	1.26
29	1,3-diaminobenzene	52.00	0.24	0.84	0.00	1.64	1.28
30	2-Methyl-4-nitroaniline	71.80	0.42	0.36	0.00	1.91	1.22
31	2,4-Dinitroaniline	118.00	0.30	0.46	0.00	1.78	1.44
32	2-Chloro-4-nitroaniline	71.80	0.46	0.30	0.00	1.84	1.41
33	2-Chloro-4,6-dinitroaniline	118.00	0.23	0.57	0.00	2.09	1.56
34	1,1'-Carbonyldiimidazole	52.70	0.00	1.25	-0.02	2.25	1.47
35	Etiracetam	63.40	0.49	1.32	0.00	1.87	1.03
36	2-Amino-4-methyl-6-methoxy-s-triazine	73.90	0.23	0.98	0.00	1.26	1.07
37	Citrazinic acid	86.60	1.56	1.22	0.00	1.61	1.23
38	2-Amino-1,3,5-triazine	64.70	0.23	0.82	-2.02	1.24	1.00
39	4-Iodoaniline	26.00	0.31	0.30	0.00	1.28	1.53
40	Imidazole	28.70	0.42	0.78	0.00	0.85	0.71
41	4-Methylimidazole	28.70	0.35	0.51	0.00	0.99	0.64
42	3,3'-Sulfonyldianiline	94.60	0.45	1.27	0.00	2.76	1.82

n_e: obtained from https://chemicalize.com/app/calculation; A, B: obtained from UFZ-LSER database.
